# Multiple organ dysfunction syndrome in critically ill children: clinical value of two lists of diagnostic criteria

**DOI:** 10.1186/s13613-016-0144-6

**Published:** 2016-04-29

**Authors:** Andréanne Villeneuve, Jean-Sébastien Joyal, François Proulx, Thierry Ducruet, Nicole Poitras, Jacques Lacroix

**Affiliations:** Division of Pediatric Critical Care, Department of Pediatrics, Sainte-Justine Hospital, CHU Sainte-Justine, Room 3431, 3175 Chemin de la Côte-Ste-Catherine, Montreal, QC H3T 1C5 Canada

**Keywords:** Critical care, Diagnosis, Intensive care, Mortality, Multiple organ failure, Paediatric

## Abstract

**Background:**

Two sets of diagnostic criteria of paediatric multiple organ dysfunction syndrome (MODS) were published by Proulx in 1996 and by Goldstein in 2005. We hypothesized that this changes the epidemiology of MODS. Thus, we determined the epidemiology of MODS, according to these two sets of diagnostic criteria, we studied the intra- and inter-observer reproducibility of each set of diagnostic criteria, and we compared the association between cases of MODS at paediatric intensive care unit (PICU) entry, as diagnosed by each set of diagnostic criteria, and 90-day all-cause mortality.

**Methods:**

All consecutive patients admitted to the tertiary care PICU of Sainte-Justine Hospital, from April 21, 2009 to April 20, 2010, were considered eligible for enrolment into this prospective observational cohort study. The exclusion criteria were gestational age < 40 weeks, age < 3 days or > 18 years at PICU entry, pregnancy, admission immediately after delivery. No patients were censored. Daily monitoring using medical chart ended when the patient died or was discharged from PICU. Mortality was monitored up to death, hospital discharge, or 90 days post PICU entry, whatever happened first. Concordance rate and kappa score were calculated to assess reproducibility. The number of MODS identified with Proulx and Goldstein definitions was compared using 2-by-2 contingency tables. Student’s *t* test or Wilcoxon signed-ranked test was used to compare continuous variables with normal or abnormal distribution, respectively. We performed a Kaplan–Meier survival analysis to assess the association between MODS at PICU entry and 90-day mortality.

**Results:**

The occurrence of MODS was monitored daily and prospectively in 842 consecutive patients admitted to the PICU of Sainte-Justine Hospital over 1 year. According to Proulx and Goldstein diagnostic criteria, 180 (21.4 %) and 314 patients (37.3 %) had MODS over PICU stay, respectively. Concordance of MODS diagnosis over PICU stay was 81.3 % (95 % CI 78.6–83.9 %), and kappa score was 0.56 (95 % CI 0.50–0.61). Discordance was mainly attributable to cardiovascular or neurological dysfunction criteria. The proportion of patients with MODS at PICU entry who died within 90 days was higher with MODS diagnosed with Proulx criteria (17.8 vs. 11.5 %, *p* = 0.038), as well as the likelihood ratio of death (4.84 vs. 2.37). On the other hand, 90-day survival rate of patients without MODS at PICU entry was similar (98.6 vs. 98.9 % (*p* = 0.73).

**Conclusions:**

Proulx and Goldstein diagnostic criteria of paediatric MODS are not equivalent. The epidemiology of paediatric MODS varies depending on which set of diagnostic criteria is applied.

**Electronic supplementary material:**

The online version of this article (doi:10.1186/s13613-016-0144-6) contains supplementary material, which is available to authorized users.

## Background

Multiple organ dysfunction syndrome (MODS), a common and deadly condition, is frequently observed in paediatric intensive care units (PICU) [1]. The pathophysiology of MODS is characterized by a severe, systemic, somewhat uncontrolled inflammatory process that leads to multiple organ or system dysfunctions [2]. There is a consensus that MODS should be diagnosed when the dysfunction of at least two organs or systems is simultaneously observed. On the other hand, there is no consensus on the criteria that must be used to diagnose the organ dysfunctions. The diagnostic criteria of MODS changed over time. A first set of diagnostic criteria was proposed in 1986 by Wilkinson [3], which was updated in 1996 by Proulx [4]. In 2005 following an international symposium on sepsis and MODS, experts presented a revised set of diagnostic criteria, authored by Goldstein [5, 6]. The goal of this 2005 definition was to “identify a reproducible assessment of organ dysfunction that allows for tracking of changes in organ function, both improvement and deterioration”.

These sets of diagnostic criteria of paediatric MODS are used in the medical literature. In this study, we determined the epidemiology of MODS, according to the sets of diagnostic criteria of Proulx [4] and Goldstein [5, 6], we studied the intra- and inter-observer reproducibility of each set of diagnostic criteria, and we compared the association between cases of MODS at paediatric intensive care unit (PICU) entry, as diagnosed by each set of diagnostic criteria, and 90-day all-cause mortality.

## Methods

### Study population

All consecutive patients admitted to the tertiary care PICU of Sainte-Justine Hospital (Montreal, Canada), from 21 April 2009 to 20 April 2010, were considered eligible for enrolment into this prospective observational cohort study. The exclusion criteria were: gestational age less than 40 weeks, age less than 3 days or more than 18 years at PICU entry, pregnancy, admission immediately after delivery. The research ethics board of Sainte-Justine Hospital approved waived consent for this study.

### Data collection

Validated case report forms were filled by PICU research assistants. Time zero for each patient was the day of PICU entry, and daily monitoring using medical chart of each individual case ended when the patient died or was discharged from PICU. We used calendar days (actuarial approach), which means that the time of data collection on day 1 and on day of PICU discharge was shorter than 24 h. Readmission within 24 h of discharge from PICU was considered as a single PICU stay. Mortality was assessed up to hospital discharge or up to 90 days post-PICU entry, whatever happened first.

### Definition of MODS

MODS is defined as the concurrent dysfunction of two or more organs or systems including respiratory, cardiovascular, haematological, neurological, gastrointestinal, hepatic and renal. In this study, we compared the diagnostic criteria suggested in 1996 by Proulx [4] and in 2005 by Goldstein [5, 6] (these two sets of diagnostic criteria are detailed in the Additional file 1). In both sets, one to five parameters are used to define each organ dysfunction, which is diagnosed if at least one criterion per organ is met. Some important differences between the two definitions should be underlined. For example, the diagnostic criteria of Proulx include gastrointestinal dysfunction, which was not retained by Goldstein. Moreover, Proulx set of criteria uses two age strata (less than 1 year old or more than 1 year old) to define low blood pressure, bradycardia and tachycardia. Heart rate is not retained in Goldstein set of criteria, but it uses six different strata of age to define normal blood pressure.

Patients with “new MODS” included (1) children with no organ dysfunction at PICU entry who developed two or more concurrent organ dysfunction while in PICU, or (2) children with only one organ dysfunction at PICU entry who subsequently developed concurrently at least another organ dysfunction. “Progressive MODS” was diagnosed when a patient with MODS at PICU entry (i.e. concurrent dysfunction of two or more organ systems) died subsequently or developed at least one additional organ dysfunction.

### Statistical analysis

Descriptive statistics included number and proportions (%), likelihood ratio, odds ratio, 95 % confidence interval (95 % CI) and mean ± standard deviation (SD).

To assess intra-observer reproducibility, a research assistant (Marianna Dumitrescu) filled twice, at least 2 months apart, the case report form of 100 randomly selected patients. In order to evaluate inter-observer reproducibility, two different observers (Marianna Dumitrescu and Marilyn Gaudreau) filled the case report form of the same 100 patients. The number of MODS identified with Proulx and Goldstein definitions was compared using 2-by-2 contingency tables. Concordance rate and kappa score (қ) were calculated to assess intra- and inter-observer reproducibility of MODS diagnosis with both sets of criteria.

Student’s *t* test or Wilcoxon signed-ranked test was used to compare continuous variables with normal or abnormal distribution, respectively. The association between MODS diagnosis at PICU entry and 90-day mortality was assessed by performing a Kaplan–Meier survival analysis. No patients were censored: all were monitored up to death, hospital discharge or 90 days post-PICU entry, whatever happened first.

Type 1 error was fixed at 5 %; *p* value <0.05 was considered statistically significant. The data were analysed using the statistical software SAS program (SAS, release 9.3, SAS Institute Inc., Cary, NC, USA).

## Results

Over the 1-year study period, there were 913 consecutive PICU admissions at Sainte-Justine Hospital. We excluded 71 because of gestational age <40 weeks (*n* = 41), because of age at PICU entry <3 days or >18 years (*n* = 40) or because the patient was admitted immediately after delivery (*n* = 1); more than one exclusion criterion was noted in some patients. Demographic information about the 842 PICU patients retained in the study is reported in Table 1.

**Table 1:** Population description

	MODS (Proulx)^a^	MODS (Goldstein)^b^	All patients^c^
	*N* = 180	*N* = 314	*N* = 842
Demographic data			
Male	98 (54.5)	168 (53.5)	434 (51.5)
Age (months)	60 ± 72	64 ± 70	72 ± 72
Severity of illness at PICU entry			
PRISM score	11.4 ± 7.8	9.2 ± 7.1	6.0 ± 5.8
Daily PELOD score	10.3 ± 9.4	8.1 ± 8.4	4.8 ± 6.8
Main cause of admission^d^			
Respiratory disease	76 (42.2)	146 (46.8)	298 (36.4)
Shock			
Hypovolemic shock	10 (5.6)	10 (3.2)	19 (2.5)
Septic shock	17 (9.6)	19 (6.1)	27 (3.2)
Haemorrhagic shock	4 (2.5)	5 (1.6)	5 (1.6)
Cardiogenic shock	13 (7.5)	13 (4.2)	15 (1.8)
Congenital heart disease	29 (16.3)	39 (12.6)	77 (9.2)
Bacterial infection	70 (39.1)	125 (39.9)	237 (28.2)
Viral infection	46 (25.8)	97 (31.1)	203 (24.2)
Trauma			
Polytraumatism	4 (2.2)	11 (3.5)	18 (2.4)
Severe head trauma	6 (3.3)	10 (3.1)	11 (1.3)
Burn	2 (1.1)	2 (0.6)	5 (0.6)
Surgery			
Post-cardiac surgery	22 (12.3)	38 (12.1)	105 (12.5)
Other surgery (planned)	17 (9.5)	33 (10.5)	146 (17.4)
Other surgery (unplanned)	14 (7.8)	24 (7.7)	63 (7.5)
Other reasons for admission	91 (50.6)	145 (46.2)	368 (43.8)
Specific treatment during PICU stay			
ECMO	7 (3.9)	7 (2.3)	7 (0.8)
Haemofiltration	6 (3.3)	7 (2.3)	7 (0.8)
Haemodialysis	10 (5.5)	9 (2.9)	15 (1.5)
At least 1 red cell transfusion	91 (50.6)	101 (32.2)	142 (16.9)

Number (%) or mean ± SD

*ECMO* extracorporeal membrane oxygenation, *MODS* multiple organ dysfunction syndrome, *PELOD* paediatric logistic organ dysfunction, *PICU* paediatric intensive care unit, *PRISM* paediatric risk of mortality

^a^MODS (Proulx): cases of MODS diagnosed during PICU stay, using diagnostic criteria advocated by Proulx in 1996 [4]

^b^MODS (Goldstein): cases of MODS diagnosed during PICU stay, using diagnostic criteria advocated by Goldstein in 2005 [5, 6]

^c^Include patients with and without MODS

^d^There were many causes of admission in some patients

### Epidemiology

Using Proulx and Goldstein diagnostic criteria, the number of patients with MODS during PICU stay was 180 (180/842 = 21.4 %) and 314 (37.3 %), respectively (Table 1). We observed new MODS in 56 (56/842 = 6.7 %) and 65 children (7.7 %) and progressive MODS in 109 (12.9 %) and 104 patients (12.4 %), respectively. The proportion of MODS diagnosed at PICU entry that developed into progressive MODS was 87.9 % (109/124) and 41.8 % (104/249) with Proulx and Goldstein criteria, respectively. The incidence rate of new and progressive MODS was 19.6 and 20.1 % with Proulx and Goldstein definitions, respectively.

### Reproducibility

Reproducibility was tested in 100 patients. The inter-observer concordance rate of MODS diagnosis was 93 % (kappa: 0.84; 95 % CI 0.72–0.95) using Proulx criteria and 93 % (kappa: 0.86; 95 % CI 0.76–0.96) using Goldstein criteria. Intra-observer reproducibility was 89 % (kappa: 0.74; 95 % CI 0.59–0.88) vs. 90 % (kappa: 0.80; 95 % CI 0.68–0.92).

### Concordance of diagnostic criteria

We calculate concordance and kappa score to estimate whether MODS or a given organ dysfunction was diagnosed twice or not diagnosed twice by the same observer in the same patients, using in the same patients the two sets of diagnostic criteria (inter-tests reproducibility). Concordance in 842 patients between Proulx and Goldstein MODS diagnosis at PICU entry was 81 % (95 % CI 79–84 %) and the kappa score was 0.49 (95 % CI 0.43–0.56) (Table 2). The concordance and kappa score for new (93 %) and progressive MODS (89 %) were high, with similar kappa scores 0.50. The concordance of the organ dysfunctions ranges from 67 % (neurological) to 99 % (renal dysfunction); the kappa scores range from 0.17 (cardiovascular) to 0.91 (renal dysfunction).

**Table 2:** Inter-test reproducibility of two sets of diagnostic criteria of MODS in 842 consecutive PICU patients

	Diagnosis of MODS or organ dysfunction in 842 patients				
	Proulx, patients^a^	Goldstein patients^b^	Concordance^c^	*p* value	Kappa score^c,d^
*MODS during PICU stay*					
1. MODS at PICU admission	124 (14.7 %)	249 (29.6 %)	81 %	<0.001	0.49 (0.43–0.56)
Progressive MODS^e^	109 (12.9 %)	104 (12.4 %)	89 %	<0.001	0.50 (0.42–0.59)
2. New MODS	56 (6.7 %)	65 (7.7 %)	93 %	<0.001	0.50 (0.39–0.61)
MODS during PICU stay (1 + 2)	180 (21.4 %)	314 (37.3 %)	80 %	<0.001	0.56 (0.50–0.61)
*Organ dysfunction during PICU stay*					
Respiratory	317 (37.7 %)	373 (44.3 %)	92 %	<0.001	0.85 (0.81–0.88)
Cardiovascular	125 (14.9 %)	15 (1.8 %)	86 %	<0.001	0.17 (0.09–0.25)
Haematological	93 (11.1 %)	122 (14.5 %)	90 %	<0.001	0.55 (0.46–0.63)
Neurological	150 (17.8 %)	428 (50.8 %)	67 %	<0.001	0.34 (0.30–0.39)
Gastrointestinal	13 (1.5 %)	Not applicable	–	–	–
Hepatic	65 (7.7 %)	107 (12.7 %)	93 %	<0.001	0.62 (0.54–0.71)
Renal	23 (2.7 %)	25 (3.0 %)	99 %	<0.001	0.91 (0.83–0.99)

95 % CI 95 % confidence interval

*MODS* multiple organ dysfunction syndrome, *PICU* paediatric intensive care unit

^a^Diagnostic criteria of MODS advocated by Proulx in 1996 [4]

^b^Diagnostic criteria of MODS advocated by Goldstein in 2005 [5, 6]

^c^Concordance and kappa score estimate whether a given organ dysfunction was diagnosed twice or not diagnosed twice by the same observer in the same patients (inter-tests reproducibility), using in the same patients the two sets of diagnostic criteria of Proulx and Goldstein

^d^According to Kramer [21], a kappa score is considered slight if <0.2, fair if between 0.2 and 0.4, moderate if between 0.4 and 0.6, substantial if between 0.6 and 0.8 and almost perfect if >0.8

^e^Progressive MODS can happen only in patients with MODS at PICU admission

### Mortality associated with MODS

**Fig. 1:**
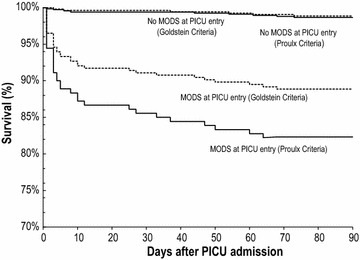
Survival analysis. Survival among 842 children with or without MODS at PICU entry, as defined by Proulx [4] or by Goldstein [5, 6]; no patient was lost or censored. There are four Kaplan–Meier curves: (1) patients without MODS, as diagnosed by the Goldstein diagnostic criteria (*upper hatched curve*); (2) patients without MODS, as diagnosed using Proulx diagnostic criteria (*upper plain curve*); (3) patients with MODS, as diagnosed by the Goldstein diagnostic criteria (*lower hatched curve*); (4) patients with MODS, as diagnosed by the Proulx diagnostic criteria (*lower plain curve*). There is a statistically significant difference between the two survival curves of patients with MODS according to Proulx and Goldstein definitions (*lowest two curves*) (*p* < 0.001). *MODS* multiple organ dysfunction syndrome, *PICU* paediatric intensive care unit

Forty-two patients (42/842 = 5.0 %) died, 28 (28/42 = 66.7 %) during their PICU stay and 41 (97.6 %) within 90 days after PICU entry (one patient died after more than 90 days). The 90-day all-cause mortality was higher when MODS was diagnosed at PICU entry using Proulx criteria (17.8 vs. 11.5 %, *p* = 0.038); likewise, the likelihood ratio of death in patients with MODS at PICU entry was higher with Proulx criteria (4.84 vs. 2.37). On the other hand, the 90-day survival rate of patients without MODS at PICU entry was similar (98.6 vs. 98.9 % (*p* = 0.73). There was a statistically significant difference between the survival curves of the two definitions for patients with MODS (*p* < 0.001) (Fig. 1).

## Discussion

### Epidemiology

MODS is a syndrome. By definition, a syndrome is a group of symptoms and signs that consistently occur together or a condition characterized by a set of associated symptoms and signs that is so common that it cannot be attributed to hazard. In theory, the diagnosis of a syndrome like MODS could be based on clinical and/or laboratory data. In practice, we do not have a pathognomonic laboratory test that can be used as a gold standard to diagnose MODS. Therefore, MODS is presently a clinical diagnosis that is based on lists of criteria.

Two sets of diagnostic criteria are currently used in the medical literature: one was published by Proulx [4] and the other one by Goldstein [5, 6]. In our hands, the incidence of MODS was 21.4 and 37.3 % with the diagnostic criteria of Proulx and Goldstein, respectively. This important difference in the incidence rate of MODS diagnosed by these two sets of criteria is probably attributable to different incidence rates of cardiovascular (14.9 vs. 1.8 %) and neurological dysfunction (17.8 vs. 50.8 %).

Our study reports many other important epidemiological data on paediatric MODS. In the literature, the prevalence and incidence rate of MODS reported in critically ill children while in PICU range from 6 to 71 % [7, 8]. Using Proulx definition, Tantalean [9] reported MODS in 56.5 % of patients during their PICU stay; 84.6 % of these MODS were present at PICU entry. Applying the same criteria, we observed a much lower prevalence (21.4 %) of MODS at PICU entry.

Using Goldstein diagnostic criteria, Typpo [10] reported a prevalence of MODS at PICU entry of 18.6 %; we noted a prevalence of 37.3 %. This difference might be attributable to different case-mix: Typpo and colleagues excluded patients younger than 1 month of age, while we did not; moreover, they excluded all patients from all hospitals where more than 10 % missing values for MODS variables were missing.

We checked whether the prevalence in PICU of the individual organ dysfunction was similar with the two sets of criteria (Table 2). Otherwise than the cardiovascular dysfunction, the prevalence during PICU stay of all organ dysfunctions was higher with Goldstein than with Proulx criteria. Using concordance and kappa score, we also checked whether both sets of criteria diagnosed the same organ dysfunction in the same patients; inter-test reproducibility was poor in cardiovascular (kappa = 0.17; 95 % CI 0.09–0.25) and neurological dysfunction (0.34; 95 % CI 0.30–0.39). This poor inter-test reproducibility is probably attributable to different definitions of cardiovascular and neurological dysfunction. The low prevalence of cardiovascular dysfunction as diagnosed with Goldstein criteria (1.8 %) is probably explained by the fact that a patient should receive a fluid bolus (≥40 ml/kg) to be considered as having a cardiovascular dysfunction. This criterion may exclude patients in cardiogenic shock or with congenital heart disease treated with inotropes for which large fluid boluses are avoided. The prevalence of neurological dysfunction was lower with Proulx than with Goldstein criteria (17.8 vs. 50.8 %); this is probably explained by more severe criteria with the former (Glasgow coma score <5 or fixed dilated pupils) than with the latter (Glasgow coma score ≤11 or acute change in mental status with a change in Glasgow coma score ≥3 points from abnormal baseline). Gastrointestinal dysfunction is only considered in the set of diagnostic criteria of Proulx. It is rare (1.5 %) and it did not contribute importantly to the observed discordance. The utility of including gastrointestinal dysfunction in MODS definition is questionable.

Diagnosis of MODS using Proulx and Goldstein criteria is not interchangeable. Weiss [11] reported a sevenfold difference in the frequency of sepsis, using different definitions. Hence, any changes to the diagnostic criteria of a syndrome like MODS or a disease like sepsis will inevitably change their epidemiology. It is not rare to read a paper where a “modified” or “adapted” definition of a set of diagnostic criteria is used; this practice must be banned unless the new definition is well validated and its epidemiology well compared to the epidemiology of previous definitions. Practitioners and investigators must carefully account for variations in diagnostic methods when reviewing and interpreting the literature, or preparing a clinical trial.

New and progressive MODS was used as a composite outcome measure in many randomized controlled trials that enrolled critically ill patients [12–14]. Leteurtre [15] determined the incidence rate of MODS in 3669 consecutive patients enrolled in two European PICUs: the incidence rate of new and progressive MODS was 5.5 and 4.7 %, respectively (total: 10.2 %). Weiss [16] reported that 30 % of children subsequently developed new or progressive MODS after sepsis recognition. In our study, the incidence rate of new and progressive MODS was 19.6 and 20.1 % with Proulx and Goldstein definitions, respectively, which is quite similar; this suggests that using one rather than the other set of diagnostic criteria of organ dysfunction would not change the sample size of a randomized controlled trial where the primary outcome measure is new or progressive MODS. On the other hand, the incidence rate of new or progressive MODS (range 10.2 and 20.1 %) is at least three times higher than the incidence rate of PICU mortality (2.7 %; site range 1.3–5.0 %) reported by Pollack in 2015 in seven American sites [17]. Thus, choosing a composite outcome that includes new and progressive MODS—progressive MODS containing all deaths—rather than mortality alone as the primary outcome measure of a randomized controlled trial conducted in PICU should decrease significantly the sample size required to complete the trial.

### Test validation

A set of diagnostic criteria is a test that is used to discriminate patients with or without a disease. The best way to validate a test is to determine its sensitivity and specificity, which required a gold standard able to classify patients in the right group (with or without the disease). However, there is presently no gold standard that can be used to differentiate patients with or without MODS. Therefore, we were unable to compare the sensitivity and specificity of the two sets of diagnostic criteria to diagnose MODS, using a gold standard. This is why this study does not tell us if the discriminative value of one set of diagnostic criteria is better that the other.

However, sensitivity and specificity are not the only parameters that can be used to validate a test: reproducibility is another important quality of a test [18, 19]. Juskewitch [20] reported an inter-observer agreement to diagnose paediatric cases of systemic inflammatory response syndrome of 88 % (95 % CI 74–94) with a kappa score of 0.75 (95 % CI 0.59–0.92), which is considered “substantial agreement” [21]. We study intra- and inter-observer reproducibility of two sets of diagnostic criteria [5, 6]. The inter-observer agreement of MODS diagnosis was 93 % (kappa: 0.84; 95 % CI 0.72–0.95) using Proulx criteria and 93 % (kappa: 0.86; 95 % CI 0.76–0.96) using Goldstein criteria. Intra-observer reproducibility was 89 % (kappa: 0.74; 95 % CI 0.59–0.88) vs. 90 % (kappa: 0.80; 95 % CI 0.68–0.92). Thus, inter- and intra-observer reproducibility of both sets of diagnostic criteria was almost similar and would be considered “almost perfect” by experts in epidemiology [21].

### Association between MODS and outcomes

A diagnosis is more useful if it is associated with predictable outcomes [22]. MODS is associated not only with short-term mortality [10, 15, 23], but also with long-term adverse events, such as poor functional outcomes [10]. MODS is diagnosed in 66–91.5 % of patients who die in PICU [9, 10]. In the literature, the mortality rate of critically ill children having MODS ranges from 10 to 57 % [8, 10]. Tantalean [9], using Proulx diagnostic criteria, reported a 41.6 % mortality rate in children with MODS. Using Proulx criteria, we report a 90-day mortality rate of 19.8 % in patients with MODS at PICU entry, while it was 11.5 % (*p* = 0.038) using Goldstein criteria. On the other hand, the risk of death was lower in patients without MODS at PICU entry, irrespective of the definition used (Fig. 1). MODS remains highly associated with mortality in critically ill children, regardless of the set of diagnostic criteria chosen.

### Strengths and limitations

The prospective design of our study is a considerable strength. Moreover, we enrolled consecutive PICU patients over a 1-year period, which enhances representativeness.

Our study has several limitations. First, one can wonder whether it is relevant to compare two different sets of diagnostic criteria that were published 10 years apart. We believe that it is relevant because the relationship between these two sets has never been evaluated. Moreover, both sets of diagnostic criteria—Proulx and Goldstein—are presently used [24–26]. We showed that these two sets do not diagnose MODS in the same patients, something that is frequently ignored. Second, we completed a single-centre study, which limits its external validity. However, when comparing severity of illness indicators and mortality rates, our PICU seems comparable to other level III multidisciplinary university-affiliated centres. Third, this study does not determine which definition—Proulx or Goldstein—should be used to study MODS in critically ill children. However, it provides information on reproducibility and likelihood of 90-day mortality that should allow investigators and practitioners to choose the most appropriate set of diagnostic criteria of MODS, given their scientific or clinical objectives.

## Conclusions

Different prevalence and incidence of MODS in different papers might be attributable to many causes: study design (retro- vs. prospective), different case-mix of patients, secular trends, detection bias, reporting bias, different sources of data and chance; it can also be attributable to the use of different diagnostic criteria [27]. Proulx [4] and Goldstein [5, 6] suggested two different sets of diagnostic criteria of paediatric MODS. These two sets are not equivalent. Both definitions have good inter- and intra-observer reproducibility, and both are closely associated with short-term mortality. However, the association between MODS diagnosed with Proulx criteria and 90-day mortality was higher than with Goldstein criteria.

The diagnostic criteria of MODS can be improved; we can look for a better alignment between diagnosis of MODS and its pathophysiology (uncontrolled systemic inflammation) and/or a better predictive value (mortality, post-PICU sequelae, quality of life). Finding a test or a group of tests that can reliably support the diagnosis of MODS would be very useful.

## Additional file


10.1186/s13613-016-0144-6 Supplementary material on the multiple organ dysfunction syndrome: sets of diagnosticcriteria suggested by Proulx et al. [4] and by Goldstein et al. [5, 6].

## Abbreviations

aPTT: activated partial thromboplastin time
ARDS: acute respiratory distress syndrome
CI: confidence interval
ECMO: extracorporeal membrane oxygenation
ICU: intensive care unit
MODS: multiple organ dysfunction syndrome
OR: odds ratio
PELOD: paediatric logistic organ dysfunction
PICU: paediatric ICU
PRISM: paediatric risk of mortality
PT: prothrombin time
