# Supplementary material for: Multiple organ dysfunction syndrome in critically ill children: clinical value of two lists of diagnostic criteria
Source: Ann Intensive Care. 2016 Apr 29;6:40. doi: 10.1186/s13613-016-0144-6 (PMC4851677; doi:10.1186/s13613-016-0144-6)
Supplement: Supplementary file 1 — 10.1186/s13613-016-0144-6 Supplementary material on the multiple organ dysfunction syndrome: sets of diagnosticcriteria suggested by Proulx et al. [4] and by Goldstein et al. [5, 6]. [file 13613_2016_144_MOESM1_ESM.docx]

# ELECTRONIC SUPPLEMENT

### Appendix 1. Definition of paediatric multiple organ dysfunction syndrome (MODS) as defined by Proulx in 1996 [4]

MODS is defined as the concurrent dysfunction of two or more systems, Each organ failure or dysfunction is defined by meeting one or more criteria of each definition^a^.

**Respiratory dysfunction**.

1. Respiratory rate >90 breaths/min (<1 yr) or >70 breaths/min (≥1 yr);
2. PaO_2_ <40 torr (5.3 kPa) in the absence of cyanotic congenital heart disease;
3. PaCO_2_ >65 torr (8.7 kPa);
4. PaO_2_/FiO_2_ <200 in the absence of congenital cyanotic heart disease;
5. Mechanical ventilation (>24 hr if postoperative).

**Cardiovascular dysfunction**.

1. Systolic blood pressure (BP) <40 mm Hg (<1 yr) or <50 mm Hg (≥1 yr);
2. Heart rate <50 or >220 beats/min (<1 yr) or <40 or >200 beats/min (≥1 yr);
3. Cardiac arrest;
4. pH <7.2 with normal PaCO_2_;
5. Continuous vasoactive drug infusion for haemodynamic support (excluding dopamine infusion ≤5 µg/kg/min).

**Haematological dysfunction**.

1. Haemoglobin <50 g/L (5 g/dL);
2. White blood cell count <3 x 10^9^/L (3 000/mm^3^);
3. Platelets count <20 x 10^9^/L (20,000/mm^3^);
4. Disseminated intravascular coagulation (PT >20 seconds or aPTT >60 seconds in presence of positive assay for fibrin-split products or D-dimers >0.5 µg/mL).

**Neurological dysfunction**.

1. Glasgow coma score <5;
2. Fixed, dilated pupils.

**Hepatic dysfunction**. Total bilirubin >60 µmol/L (3 mg/dL).

**Gastrointestinal dysfunction**. Gastroduodenal bleeding and one of the following criteria believed to be the result of gastroduodenal bleeding by the treating physician:

1. Drop in the haemoglobin level ≥20 g/L (≥2 g/dL) over 24 hours;
2. Blood transfusion;
3. Hypotension with blood pressure <3^rd^ percentile for age;
4. Gastric or duodenal surgery;
5. Death.

**Renal dysfunction**.

1) Serum urea nitrogen (BUN) value >36 mmol/L (>100 mg/dL);

2) Serum creatinine >177 µmol/L (>2.0 mg/dL) without preexisting renal disease;

3) Dialysis and/or haemofiltration.

aPTT: activated partial thromboplastin time; PT: prothrombin time.

^a^Non-survivors must be considered to have all organs failing on date of death.

### Appendix 2. Definition of paediatric multiple organ dysfunction syndrome (MODS) as defined by Goldstein in 2005 [5, 6]

MODS is defined as the concurrent dysfunction of two or more systems. Each organ failure or dysfunction is defined by meeting one or more criteria of each organ or system.

**Cardiovascular dysfunction**

Despite administration of intravenous fluid bolus ≥40 mL/kg in 1 hour:

1) Decrease in blood pressure (hypotension) <5^th^ percentile for age or systolic blood pressure <2 SD below normal for age ^a^ **OR**

1. Need for vasoactive drug to maintain blood pressure in normal range (dopamine >5 μg/kg/min or dobutamine, epinephrine, or norepinephrine at any dose) **OR**
2. Two of the following
3. Unexplained metabolic acidosis: base deficit >5.0 mEq/L
4. Increased arterial lactate >2 times upper limit of normal
5. Oliguria: urine output <0.5 mL/kg/hr
6. Prolonged capillary refill: >5 seconds
7. Core to peripheral temperature gap >3°C

**Respiratory dysfunction ^b^**

1. PaO_2_/FiO_2_ <300 in absence of cyanotic heart disease or preexisting lung disease **OR**
2. PaCO_2_ >65 torr or 20 mm Hg over baseline PaCO_2_ **OR**
3. Proven need ^c^ for >50% FiO_2_ to maintain saturation ≥92% **OR**
4. Need for non-elective invasive or non-invasive mechanical ventilation ^d^

**Neurological dysfunction**

1. Glasgow coma score ≤11 **OR**
2. Acute change in mental status with a change in Glasgow coma score ≥3 points from abnormal baseline

**Haematologic dysfunction**

1. Platelet count <80,000/mm^3^ (<80 x 10^9^/L) or a decline of 50% in platelet count from highest value recorded over the past 3 days (for chronic haematology/oncology patients) **OR**
2. International normalized ratio (INR) >2

**Renal dysfunction**

Serum creatinine ≥2 times upper limit of normal for age or 2-fold increase in baseline creatinine

**Hepatic dysfunction**

1. Total bilirubin ≥4 mg/dL (not applicable to newborn) **OR**
2. Alanine transaminase (ALT) 2 times upper limit of normal for age

^a^Systolic blood pressure < 2 SD for age: 0 day – 1 week of age, < 59 mmHg; 1 week – 1 month, < 75; 1 month – 1 year, < 75; 2 – 5 years, < 74; 6 – 12 years, < 83; 13 – < 18 years, < 90 [5, 6].

^b^Acute respiratory distress syndrome (ARDS) must include a PaO_2_/FiO_2_ ratio ≤200 mm Hg, bilateral infiltrates, acute onset, and no evidence of left heart failure. Acute lung injury (ALI) is defined identically except the PaO_2_/FiO_2_ ratio must be ≤300 mm Hg.

^c^Proven need assumes O_2_ requirement was tested by decreasing flow with subsequent increase in flow if required.

^d^In postoperative patients, this requirement can be met if the patient has developed an acute inflammatory or infections process in the lungs that prevents him or her from being extubated.
